# Building Blocks of Virtuous Science Communication: Grant Funding, Policy Making, and Public Engagement

**DOI:** 10.1089/dna.2021.0523

**Published:** 2022-01-12

**Authors:** Anna Caballe, Martino Bardelli

**Affiliations:** ^1^ Oxford Nanoimaging (ONI), Oxford, United Kingdom.; ^2^ Department of Biochemistry, University of Oxford, Oxford, United Kingdom.

**Keywords:** funding, public engagement, policy, science communication

## Abstract

Grant writing, science policy and public engagement (PE) activities are forms of science communication, and all are essential for research to function and benefit our society. Scientists rely on competitive grant funding to finance their research; this is an opportunity for researchers to communicate their scientific vision and engage funders, major players in defining how public funds and government policies are prioritized to drive research and innovation. PE is often still seen as a box-ticking or persuasion exercise, yet establishing the right communication channels with the public is pivotal for a scientist's job to impact society. We believe that evidence-based communication is becoming essential in a world dominated by an excess of information, where verified sources are under pressure. Support in the form of dedicated training and allocated resources, particularly for early career researchers, can help establish what we describe as a long-lasting virtuous circle, in which public funds are spent effectively toward scientific advances that the public and policy makers can embrace.

## Introduction

Research and innovation (R&I) are integral to the mission of most leading universities in the United Kingdom and worldwide. Funding for research activities in academic institutions is provided by different sources. Most commonly, funding springs from government-backed awards to support the research infrastructure and/or competition-based project funding from research councils or nonpublic organizations. Such organizations include for example charities, commercial entities, national academies, private trusts or foundations, the European Commission, or the National Institutes of Health.

The term “grant” is frequently used by researchers to refer to the competitive funding granted by any of these institutions, and how to secure them is one of the main worries for emerging scientists. Alternatively, core funding (referred sometimes as “hard money”), which guarantees the researcher's base salary and a package to cover some laboratory costs, is generally institute or country specific. Although core funding is provided in a noncompetitive manner to individuals in those institutions, it is still obtained through government-derived resources.

Fundamental research and development (R&D) can often be overlooked or misunderstood by funders and members of the public, as its impact is less tangible and its long-term impact is hardly measurable. Translational research projects are usually easier to rationalize by researchers to justify the support from funders, as they provide more immediately visible outcomes and impacts. There is, however, evidence that long-term investment in R&I drives growth of a country. Data from U.K. Research and Innovation (UKRI), the umbrella institution englobing all primary U.K. Research Councils, showed that each £1 of public R&I investment on average generated at least £7 of net benefit to the U.K. economy (UKRI, 2020). Nevertheless, it can be difficult for policy makers and members of the public to value the long-term benefits of R&D.

In our opinion, funding bodies have the job and responsibility to support both research projects with clear objectives and applications, and early R&D or “blue-sky” projects. Similarly, other stakeholders, such as governments and politicians, play a key role in allocating the necessary resources and budgets for R&I. Last, but not least, the public can and should also have a voice in how public funds are used to finance research projects and get a clearer understanding on the value that R&I bring to society in the short and long term.

In this opinion piece, we discuss grant funding allocation, the challenges facing early career researchers, and the need for better public and political engagement to create a constructive cycle of information around how R&I benefits society. We propose how this virtuous cycle can be better promoted, based on our perspective and experiences as early -career scientists in the academic and the small biotech environments that regularly engage in science communication activities.

## The Road to Grant Funding Awards

Grant applications are laborious, notoriously competitive, and usually awarded based on merit (mostly on publication record, although new initiatives are being explored as discussed hereunder), which tends to favor established researchers. It is encouraging that some funders have specific awards for early career researchers and/or weigh applications to account for different levels of seniority. Both early career and established researchers go through the same process: from research proposal submission to addressing assessors' comments or a panel interview, to waiting for months for the outcome.

The more experienced a researcher, the better he or she can master the art of grant writing (and storytelling). The process needs to be thorough as it uses public or charitable funds, and the funding body has to ensure that the best projects are selected, with proposals being ambitious and innovative yet feasible. Researchers also play an active role in the assessment process, as peer reviewers, participating in panels and on advisory boards for funding bodies.

However, the entire process of grant funding requires a significant time investment away from research, making the process unnecessarily cumbersome and somewhat inefficient. Improvements are necessary to streamline the process and provide special support to early career scientists, while maintaining rigor when awarding funds. Institutional core funding can provide an alternative approach to highly competitive individual grant allocation, and has been suggested to lead to more efficient spending of public funds (Sandström and Van den Besselaar, 2018).

Different factors determine which researchers are awarded research grants. For academic institutions, allocations are made on the basis of academic excellence and impact. For commercial entities, this can mean previous funding success, the expected commercial outcomes and impact. Impact can be measured in different ways, with scientific publications being the main output considered, particularly for academic researchers. This can be problematic as bibliometric studies in various fields have shown that the “top” journals (those with high number of citations and most prestige) are heavily dominated by research produced in a small number of countries, mostly the United States and the United Kingdom (Mason and Merga, 2021).

Furthermore, a report suggested that some of the most relevant international funding schemes are affected by institutional bias, with funding panels more likely to give prestigious European Union early career grants to applicants connected to the institutions of some of the panelists (Kwon, 2021). In the United Kingdom, funding bodies have a shared policy for research assessment that is informed by the Research Excellence Framework (REF), which aims to provide accountability for public investment in research, to produce evidence of the benefits to the economy and society, and provides benchmarking information to inform the selective allocation of funding for research (REF, 2021).

Although these shared policies can be beneficial (Sandström and Van den Besselaar, 2018), they often add to the workload of researchers, which can be disadvantageous for those in smaller institutions, where less core support is available. In our opinion, funders have the responsibility to make sure that innovative research projects and excellent scientists have the same chances of being funded regardless of the institution they work in. This will likely entail the establishment of new methods to measure research impact and scientific excellence beyond the sole use of publications and impact factors. In recent years, additional impact activities such as collaboration, teaching, public engagement (PE), or contribution to policy articles are being considered, yet standardized metrics that can be used worldwide have not been agreed.

Given the current competitive landscape for access to funding, for many researchers getting funded is the exception, not the rule. The aggregate success rate for research grants at the U.S. National Institutes of Health in 2017 was only 20.5%. At the biomedical research funder Wellcome, ∼50% of applications make it through the preliminary stage and, of those, ∼20% are funded (Crow, 2020). The high chances of rejection and repeated unsuccessful applications can be heart breaking for researchers who are trying to establish themselves as group leaders. Very frequently these researchers depend on grants to keep their laboratories running and pay for salaries, which adds significant strain and creates less secure working atmospheres, sometimes leading to devastating consequences (Nature Editorial, 2020).

Core funding can provide a mean to remove direct funding pressures on individual researchers, allowing more time for research, longer term planning, and to explore research avenues of potentially less immediate impact. Although in many cases the pressure is transferred from the individual researcher to the institution to secure institute-wide financing, institutes are in a position to create specialized units that provide better and more efficient strategies to secure funding and to create links with funders, policy makers and society as a whole. This can be extremely helpful to help set up early career researchers for success.

Although we are focusing on funding for academic researchers, it is worth mentioning that several funding bodies provide funding to non-academic institutions, such as start-up companies, small biotechs, or even larger commercial institutions. In 2019–2020, UKRI invested a significant £1.6 billion in cutting-edge innovation to address major industrial and societal challenges, providing innovation grants to businesses of all sizes to commercialize the best ideas. That same period, they invested £3.28 billion in pioneering ideas through research council programs and funding for research at universities (UKRI, 2020).

Funding bodies usually allocate separate budgets for company-driven projects, which are more focused on commercialization, yet these also involve the participation of academics in the form of expert reviewers to help assess applications. Although some could think that companies have a stronger lobbying power to argue for funding, we believe this is likely compensated by the lobbying power of large universities. It is our opinion that funding bodies must ensure, in any case, that curiosity-driven research prevails and funding is not solely focused around short-term economic outputs.

## How Funding Roadmaps and Science Policies Drive Research

All funding bodies have roadmaps and missions detailing the priorities for coming years and the types of projects in their portfolio. In our experience, these are usually based on prevalent diseases and issues society faces. It can, however, hinder more specialized areas of research or emerging fields that are not considered areas of major interest or “hot topics,” as scientists may bias their research focus to ensure higher funding success. It is important that scientists approach funders and create a connection to better align project proposals to maximize funding success and make portfolios more inclusive. This may be less accessible to early career researchers and perhaps funders should find new ways to connect with them; with some initiatives starting to emerge to get researchers involved in policy development (UKRI, 2021).

Although “hot topics” can change over the years, the COVID-19 pandemic in 2020 showed that funding bodies can rapidly shift gears and work at a faster pace to grant funding to address immediate challenges. On the downside, this situation posed a threat to academics not working on COVID-19 projects, left with uncertain project timelines, or having to reinvent their research to fit into the COVID-19 scope. Funders face the challenge of constantly having to reassess their funding priorities and to adapt to the emerging needs of research and of society.

The COVID-19 world pandemic also highlighted the need to invest in research. Initial promises by governments have not necessarily materialized, however, and cuts to research budget allocation have been announced in the United Kingdom and elsewhere, justified by the economic difficulties that countries face postpandemic. U.K. universities recently made an urgent plea to cease threats of steep research funding cuts from the treasury, which would cost the sector £1 billion and imperil thousands of jobs (Adams, 2021).

**FIG. 1.**
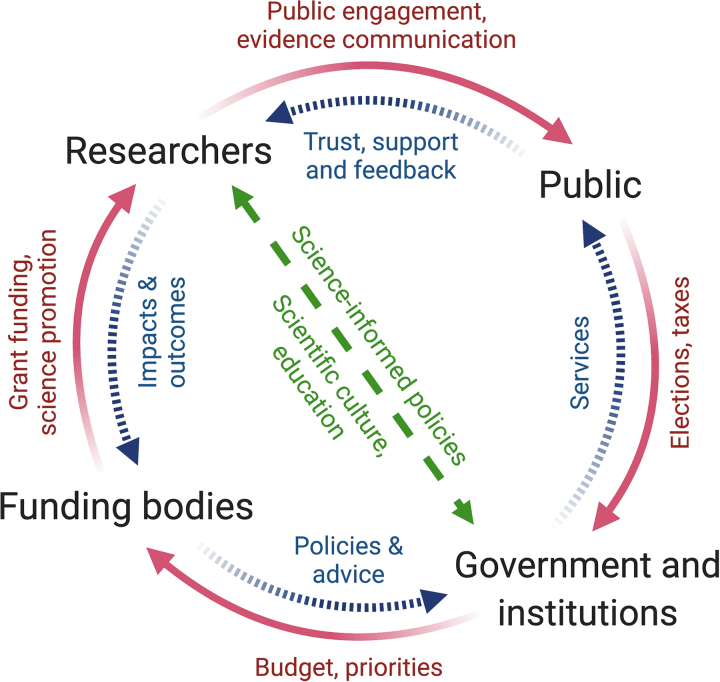
Establishing a virtuous circle in science communication. Researchers, funding bodies, government and the public all contribute to a virtuous circle in which public funds are spent effectively toward the benefit of society. Effective communication is used to build mutual interactions, understanding and trust, for public and policy makers to embrace scientific advances.

This came after UKRI announced in March 2021 that the universities' budget for international development projects had been cut almost in half, after the U.K. government's decision to shrink its international aid budget. Keeping politicians engaged with research can help overcome some of these barriers around funding allocation for R&D. Both scientists and the government need to explore new initiatives to strengthen the bond between scientists and policy makers, which form part of a crucial circle in science communication (Fig. 1, further explained hereunder).

## Public and Political Engagement Require Evidence-Based Communication

PE is defined as “the myriad of ways in which the activity and benefits of higher education and research can be shared with the public. Engagement is by definition a two-way process, involving interaction and listening, with the goal of generating mutual benefit” (NCCPE, 2021). PE allows a wide variety of stakeholders (from children to policy makers patient advocacy groups or NGOs) to create a common language and understanding of the problems, the opportunities and controversies at the forefront of scientific research.

Effective PE is a two-way process: the public benefits from an increased ability to understand scientific problems, while the results of the engagement are fed back to the researchers and into the research, benefitting all parties involved. PE also helps researchers put their research into a wider context and improve their communication skills. Nowadays, virtually all funding bodies promote some form of PE. For example, the European Commission's Horizon 2020 scheme integrated a responsible R&I approach, an inclusive paradigm to “better align the process and outcomes of research and innovation, with the values, needs and expectations of European society” (European Commission, 2014).

Universities and research institutes also actively promote PE activities, such as science festivals and open doors days, or through informal science learning centers or school visits, and some require researchers to use contract time to engage in outreach activities (i.e., University of Oxford). However, individual initiatives might have limited impact, and better coordinated and more “professionalized” science communication should ensure that measurable outputs are achieved and made public, for both researchers and public to understand what effective science communication entails (Ziegler *et al.*, 2021).

Effective communication also provides the basis for scientists to engage with policy makers. A first step is identifying key challenges that the world is facing and promoting evidence-based policy making among the stakeholders involved. Although the importance of evidence-based policies is recognized across political groups and processes have been implemented, some difficulties remain (Gerber, 2020). The timetables of science and politics are vastly different, political decisions are often driven by values rather than evidence, and policies do not always have measurable outcomes (Rutter, 2012).

Independent transparent external institutions that are connected to both the scientific and political communities can bridge this gap. An example in this direction is the MP–scientist partnership promoted by the Royal Society since 2001, which helps to create a common language and establish personal trust relationships to promote evidence-based policies. Some researchers also have roles in advising policy makers, for instance, as chief scientific advisers, although once again these tend to exclude early career researchers and efforts should be made to change this pattern.

We live in a time where we are constantly inundated with new information. This “infodemics” is plagued by the rapid and wide spread of false news or unverified information (not only in science), forcing the scientific community to find ways to effectively engage the widest possible audiences. Because it is generally accepted that evidence should guide decisions, evidence communication should increasingly become an integral part of a researcher's career. Key to a successful engagement is trust; scientists need to rely on their expertise, but also require the public to perceive that they are being offered an honest message conveyed with good intentions (Blastand *et al.*, 2020). Rather than cherry-picking the most persuasive data, it is crucial to provide the full picture, being clear on the uncertainty of the data available and highlighting the unknowns.

Recognizing the limitations of data and the ability to admit being wrong are also important. Communication strategies widely employed by the media and politics, which rely on persuasion and confirmation bias rather than trust, are unlikely the best strategy moving forward. Effective science communication and its systematic evaluation will help create more knowledgeable audiences (Fischhoff, 2019; Ziegler *et al.*, 2021), not persuaded and led to a particular opinion, but able to understand the arguments and evidence provided to reach its own conclusions.

Making science more accessible to audiences will help make it more diverse and inclusive, and can lead to a more science-conscious society that encourages informed decision making at all levels, from government to communities to individuals (Fig. 1). Deploying creative and engaging communication strategies can make science more approachable and help reach new audiences; starting with younger audiences could help change the current paradigm, which would ensure a sustainable long-term model.

## Future Perspectives: Building a Virtuous Science Communication Circle

Writing grants, PE activities and involvement in science policy are forms of science communication, and all are essential for research to function and to benefit our society as described in this article. R&D funders influence how and which science is carried out; the results of research, in turn, drive innovation; and the engagement of researchers and innovators with the wide public and policy makers defines the priorities for the prosperous development of society, which will influence the allocation of funds to R&I, closing this important circle (Fig. 1).

We need to strive to make this a sustainable virtuous circle, in which public funds are spent appropriately and effectively, in which rigorous yet exciting and ambitious research is carried out, and in which scientists (both in academia and in the private sector), general public, and policy makers have a relationship of mutual understanding and trust. With increasing economic pressure on funding availability and the ongoing infodemic, it is imperative that early career scientists are offered the opportunity and, crucially, the time and resources to develop their science communications skills. For instance, dedicated science communication jobs should be further supported to enable scientists to focus on research and ensure science is communicated effectively across society. This would enable early career researchers to become valuable members not only of the scientific community but also of society as a whole, and make it easier for them to participate in and nurture this virtuous circle.
